# A Robust Design-Based Expert System for Feature Selection and COVID-19 Pandemic Prediction in Japan

**DOI:** 10.3390/healthcare10091759

**Published:** 2022-09-13

**Authors:** Chien-Ta Ho, Cheng-Yi Wang

**Affiliations:** Graduate Institute of Technology Management, National Chung Hsing University, 145 Xingda Rd., Taichung City 402, Taiwan

**Keywords:** artificial intelligence, expert system, robust design, feature selection, COVID-19, disease prediction, genetic algorithm, healthcare

## Abstract

Expert systems are frequently used to make predictions in various areas. However, the practical robustness of expert systems is not as good as expected, mainly due to the fact that finding an ideal system configuration from a specific dataset is a challenging task. Therefore, how to optimize an expert system has become an important issue of research. In this paper, a new method called the robust design-based expert system is proposed to bridge this gap. The technical process of this system consists of data initialization, configuration generation, a genetic algorithm (GA) framework for feature selection, and a robust mechanism that helps the system find a configuration with the highest robustness. The system will finally obtain a set of features, which can be used to predict a pandemic based on given data. The robust mechanism can increase the efficiency of the system. The configuration for training is optimized by means of a genetic algorithm (GA) and the Taguchi method. The effectiveness of the proposed system in predicting epidemic trends is examined using a real COVID-19 dataset from Japan. For this dataset, the average prediction accuracy was 60%. Additionally, 10 representative features were also selected, resulting in a selection rate of 67% with a reduction rate of 33%. The critical features for predicting the epidemic trend of COVID-19 were also obtained, including new confirmed cases, ICU patients, people vaccinated, population, population density, hospital beds per thousand, middle age, aged 70 or older, and GDP per capital. The main contribution of this paper is two-fold: Firstly, this paper has bridged the gap between the pandemic research and expert systems with robust predictive performance. Secondly, this paper proposes a feature selection method for extracting representative variables and predicting the epidemic trend of a pandemic disease. The prediction results indicate that the system is valuable to healthcare authorities and can help governments get hold of the epidemic trend and strategize their use of healthcare resources.

## 1. Introduction

Infectious diseases, if not effectively monitored and controlled, often result in mass human infections and pose risks of mass mortality, economic recession, and depletion of medical resources. For instance, coronavirus disease 19 (COVID-19) first broke out in late 2019 in Wuhan, Hubei Province, China [1,2], and then spread rapidly around the world. It was soon recognized as a global pandemic by the World Health Organization (WHO). In the next year, COVID-19 began to cause enormous impacts across the world. As of 2022, COVID-19 continues to spread aggressively from country to country, causing not only over 500 million sick people but also 6 million deaths. The unpredictable nature of COVID-19 has placed a great deal of pressure on governments to set up policies to curb the spread of the epidemic, and it is likely to cause medical resource depletion [3]. Moreover, COVID-19 has also had many negative effects on the global economic environment [4].

Since the Omicron variant began to sweep the world, the COVID-19 pandemic has intensified in many countries due to the high transmissibility of this variant. Despite the emergence of vaccines approved by emergency-use authorization (EUA), because human knowledge of this virus is still insufficient, many people are dying from it every day. In 2022, WHO once again warned the world that the spread of COVID-19 should be closely monitored to prevent a simultaneous increase in the number of moderate and severe cases and the number of deaths as the virus continues to mutate. WHO also suggested that all governments should adjust management policies and quarantine measures for severe cases in a timely manner. Therefore, health authorities’ ability to get hold of the development trends of the pandemic is particularly important.

Nowadays, the main tasks of healthcare management authorities in epidemic prevention include infection prevention, spread prediction, infection control, treatment of confirmed cases, and mortality reduction [5]. Until a highly effective method of eliminating this virus is found (including the improvement of vaccines), monitoring the spread trends and reducing the mortality of COVID-19 is a priority in epidemic management for governments seeking to maintain economic activity during the pandemic [6,7]. The prediction of the epidemic trend is linked to the government’s epidemic prevention policies [8,9,10]. How to effectively get hold of the changes in the number of deaths is an imperative task for health authorities because it is conducive to the deployment of medical resources and the improvement of healthcare policies. Therefore, developing a support system that can capture the trends of COVID-19 has become an emerging area of research.

The recent years have seen a substantial development of expert systems and publication of outstanding research findings about such systems in all fields. A human expert can quickly find feasible solutions to a new problem based on his or her experiences that accumulate over time. The introduction of the LISP programming language by John McCarthy in 1960 ushered in the development of research on expert systems [11]. Early expert systems can be represented by the problem-solving model proposed by Feigenbaum et al. in 1970 to determine the structures of chemical molecules [12]. This type of expert system is a rule-based reasoning system that can be applied to disease diagnosis. Due to certain limitations, this type of expert system soon hit a bottleneck in its development. For example, rule-based reasoning systems require the establishment of very complicated conditional formulas, where the cause–effect relationships are highly restrictive, so they are less flexible, and the cross-references between cases may be easily ignored. Hence, there is still a gap in decision-making behavior between expert systems and real human experts. Fortunately, with the advancement of computer science and the extensive use of personal computers, scholars have begun to promote new techniques of expert systems. For instance, Aliev et al. [13] proposed an if–then rules-based fuzzy technique for reasoning with imperfect information and applied it to evaluate job satisfaction and students’ educational achievement related to psychological and perceptual issues. Tang and Pedrycz [14] demonstrated the stability of an expert system. They investigated the oscillation-bound estimation of perturbations for Bandler–Kohout subproduct (BKS) and constructed upper and lower bounds of BKS output deviation derived from the simple perturbation of the input set.

Among the numerous techniques of expert systems, artificial intelligence systems (AIs) have received particular attention from researchers [15,16,17]. AIs are algorithms developed to mimic the operation and behavioral patterns of living organisms. They process information based on past experiences. Through improvements, AIs can more efficiently enhance the learning performance of expert systems and expand the scope of problem search, gradually pushing the decision-making ability of expert systems closer to the level of human experts. As expert systems can be integrated with various types of AIs, many cross-disciplinary applications have been attempted in areas such as clinical, healthcare, environmental, and industrial [18,19,20,21,22]. These applications have also contributed to the flourishing development of expert systems. AIs use datasets to train a model and create an input–output mapping. This type of operation makes AIs highly appropriate for application in data mining, knowledge engineering, medical assessment, diseases prediction, etc. [15,16,18,23,24].

In the last decade, big data analysis was a new line of research. Advancements in this area of research have contributed to the growth of expert systems. With the development of data mining, many big data-driven expert systems have been proposed [25,26,27]. This suggests that big data can widen the search scope of expert systems and also improve their training performances.

Due to the outstanding contribution of AIs to various research fields, it has also received attention in the field of healthcare. For instance, Malki et al. [28] developed a supervised decision tree model to predict the spread of COVID-19 infection in many countries. Khalilpourazari and Hashemi Doulabi [29] designed a hybrid reinforcement training-based framework to predict the COVID-19 pandemic and help policy makers to optimize the use of healthcare system capacity and resource allocation. Alam et al. [30] developed a disease diagnosis using the Internet of Things (IoT) integrated with a fuzzy inference system to diagnose various diseases.

Recent research has shown that feature selection is the most representative technique of expert systems. Expert systems usually rely on supervised learning. They need to be given a set of training data to learn the relationship between “input features” and “outcomes” [31,32].

Feature selection is a technique for extracting relevant features in data. The basic concept of feature selection is to find distinctive case features in order to enhance the learning efficiency of the expert system [33,34].

If the system can omit certain unnecessary features, it can reduce the data comparison time and achieve a higher accuracy. The main advantage of feature selection lies in its ability to adopt supervised or hierarchical feature extraction algorithms to replace manual ways [31]. The growth of feature selection (including feature extraction) is manifested in the fruitful results of recent research. The outcomes derived from a large amount of data, in particular, have drawn the attention of experts across all fields [27,32]. However, the goal of feature selection for expert systems is to learn autonomously from a large amount of data to create a better model with better training results. In addition, the benefit of using supervised feature selection in an expert system is that the system model can autonomously extract appropriate features and define the recognition or prediction result for each instance.

In an early application of feature selection, Siedlecki and Sklansky [35] used genetic algorithms (GAs) to deal with large-scale feature selection. They attempted to design a set of automatic pattern classifiers. Feature selection could help the system extract the features of patterns suitable for recognition and then deliver the selection results to the system for prediction.

As to the applications of feature selection in recent years, Lin et al. [36] proposed a technique using layered genetic programming for feature extraction to deal with the problem of optimizing the classification of data into two groups. Here, the concept of “feature extraction” refers to transferring the good features obtained in each evolution to the GA processing of the next layer, in order to achieve hierarchical optimization. Their experimental results confirmed that this technique can enhance the problem-solving performance of GAs. Quan and Ren [37] proposed a method of product feature extraction for feature-oriented opinion determination. The feature extraction technique was applied to deal with opinion mining and perform sentiment analysis for product improvement. They showed a high applicability of feature selection using comparative domain corpora.

Zhang et al. [38] proposed an ant colony algorithm-based feature selection method for intelligent fault diagnosis of rotating machinery using a support vector machine. Some scholars have applied GAs as a data mining technique to extract informative and significant features in breast cancer diagnosis [39,40]. In addition, GA-based feature selection methods can deliver a better performance [39]. Gokulnath and Shantharajah [41] employed a GA as an optimization function based on a support vector machine (SVM) for heart disease diagnosis. Khan et al. [42] proposed a hybrid feature selection and reduction scheme for selecting the high discriminative characteristics in hypertension features. Kwon et al. [43] employed feature selection methods to support the prediction of osteoporosis. They conducted a comparison of machine learning with different models and found that features selected by “survey+checkup” led to a better prediction accuracy than survey or checkup only. Moreover, machine learning could achieve good performance in disease prediction. More recent studies of expert systems have empirically demonstrated the effectiveness of applying feature selection in disease prediction, equipment examination, and mental state prediction [43,44,45].

A feature-selection-based expert system is a smart technique that can be used for describing structured data. It can converge in its own database by inputs of specific structures, so it can accept a wide range of real cases. With this characteristic, feature selection is most appropriate for experiments that involve observations with an expert system [28,29]. As the development trend of a pandemic disease is the result of a features-and-effect phenomenon that evolves over time, feature selection is a very suitable solution for the prediction of epidemic trends. A comparison of previous research on feature selection for prediction is presented in Table 1.

Previous studies have shown that it is not easy to get impressive learning results from expert systems [46,47,48,49]. Oftentimes, it is necessary to repetitively adjust and test the parameters of the system. This procedure is very time-consuming and will increase the cost of system modeling. In addition, the obtained parameter values cannot always guarantee good prediction performance in the future. The abovementioned situation reduces the robustness of the expert system. Moreover, when building a pandemic prediction system, it is necessary to adjust the system parameters whenever needed. In other words, system adaptability and reliability are also of high importance. Therefore, an effective and stable system building method must be developed so as to exploit the excellent performance of expert systems. This study aims to fill two major gaps in the literature: Firstly, the extant research of pandemics lacks studies on expert systems with a robust predictive performance. Secondly, little research has attempted to investigate feature selection methods for predicting the epidemic trend of a pandemic disease.

In this paper, a modified expert system, called the robust design-based expert system, is proposed to address the above issues. In addition, a genetic algorithm (GA) framework and the Taguchi method are integrated into the system to optimize the performance of the system. A good system configuration can not only increase the system’s prediction accuracy but also ensure the stable quality of the system. Features selected by the system can support inferences of epidemic trends. Finally, the feasibility and efficiency of the proposed system is verified using COVID-19 as an example.

## 2. The Expert System with Robust Design

### 2.1. System Architecture

**Figure 1:**
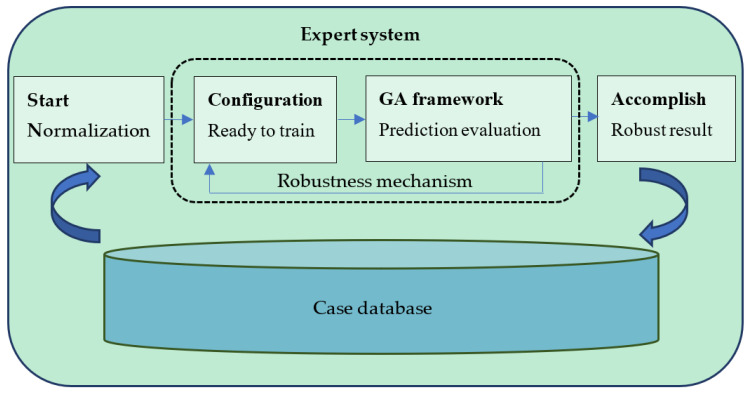
Architecture of the robust design-based expert system.

**Figure 2:**
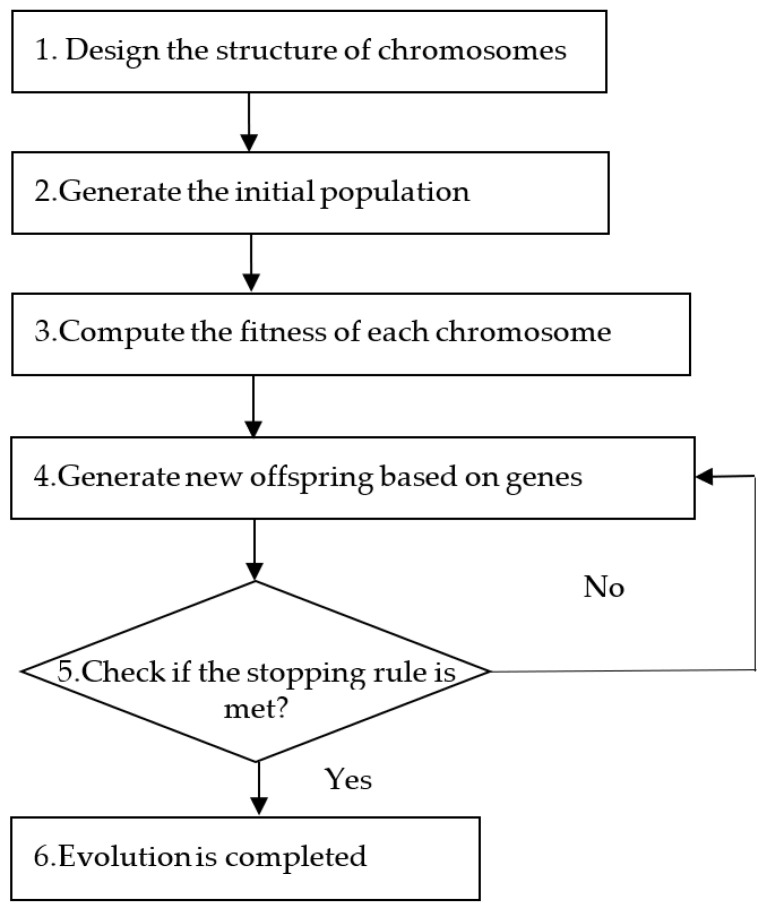
GA framework of the system.

In this study, a robust design-based expert system is proposed for pandemic prediction. The system architecture is shown in Figure 1. The operational steps of the expert system are as follows: First, the dataset is imported from the case database and normalized. Later, systematic training with the selected parameter levels is conducted. The system will learn the best pattern of features from the dataset, meaning that the system will obtain a feasible solution from the genetic algorithm (GA) framework (see Figure 2). However, this solution does not represent a robust solution of the system under different configurations. The system will repeatedly execute the procedure under different configurations through the robustness mechanism until all the runs have been completed. After all the runs have been completed, a robust result can be obtained.

### 2.2. Optimization of the System

**Figure 3:**
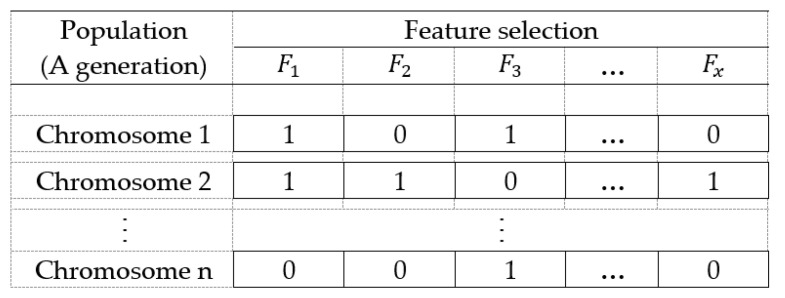
Structure of chromosomes.

To enhance the predictive performance of the proposed system, this study applies a genetic algorithm (GA) to feature selection. GAs have been widely used as a means to optimize expert systems [34,35,36]. It is an optimizing technique that mimics the evolutionary process of biological chromosomes. Based on the concept of genetic evolution, it repeatedly searches for feasible solutions in order to find an optimal solution to the given problem. The operating process of the GA is briefly explained as follows:

First of all, the GA stochastically generates an initial set of feasible solutions (called the initial population), in which each feasible solution is called chromosome and coded by a value of 0 or 1 (see Figure 3). Later, the fitness of each feasible solution is computed. The fitness function can be customized by users. A higher fitness value usually indicates a better solution. In the optimization of an expert system, the fitness function is usually defined as the accuracy of the inferential result.

Next, the GA uses the genes in the chromosomes to compute the next generation. The proposed system adopts the GA as a predictor because of its evolution mechanism including selection, crossover, and mutation. These mechanisms can help the system achieve a high prediction accuracy. Selection decides which chromosomes can survive or should be eliminated; crossover is used to exchange partial sections of the chromosomes among parents to create the chromosomes of the next generation. Finally, mutation selects one gene from chromosomes for mutation. The probability of mutation is usually very low. Through repetitive executions of the above genetic operation, offspring with better fitness can be generated, and this operation stops when the stopping rule is met.

The GA framework designed for this system is as illustrated in Figure 2. The proposed system applies the GA to select features in the dataset. This training procedure consists of six steps as explained below:

**Step 1**. Design the structure of chromosomes

In order to obtain an optimal combination of features, we encode feature selection in chromosomes using 0 or 1, as shown in Figure 3. For instance, “0” denotes the corresponding item is unselected, while “1” denotes the item is selected.

**Step 2**. Generate the initial population

Before execution of the genetic algorithm, the system has to generate an initial population comprising n chromosomes, each containing randomly generated parameter values. Each chromosome represents a possible solution (the initial feature selection). Given a total of x features, each chromosome is represented by x genes, and each generation has n chromosomes. Through evolution from one generation to the next of each generation, a better solution can be progressively obtained.

**Step 3**. Compute the fitness of each chromosome

To compute the fitness of each chromosome, we divide the case dataset into two subsets, including a training dataset and a test dataset. The training dataset is the main dataset for training the expert system, whereas the test dataset provides the subject to be tested by the expert system. The training dataset is larger than the test dataset.

For any chromosome $i\;\left(i=1,2,\ldots ,n\right)$, some features in both the training dataset and the test dataset need to be removed, weakened, or reinforced according to the set of features stored in the chromosome. Assume that $Train_{i}$ and $Test_{i}$, respectively, denote the modified training dataset and the modified test dataset. The fitness of chromosome *i* can be computed through the following steps:

(1) Compute the predicted level (*PL*) for each case in the test dataset. For each case t  in the test dataset, we apply the nearest neighbor method to find the most similar case in the training dataset $Train_{i}\;$ to predict the level of this case (the level is represented by $PL_{t}$ ). The similarity between cases is measured using Euclidean distance.
(2) Compute the fitness of chromosome $i\;\left(i=1,2,\ldots ,n\right)$. The fitness of chromosome i can be expressed using the following function:

(1) $$fitness\left(chromosome\;i\right)=\overset{D}{\underset{t=1}{\sum }}\frac{match_{t}\left(Train_{i},Test_{i}\right)}{D}$$

where D denotes the number of cases in $Test_{i}$; $match_{t}$ indicates whether the predicted level ($PL_{t}$) matches the actual level ($AL_{t}$). If $PL_{t}\;$=$\;AL_{t}$, $match_{t}\left(Train_{i},Test_{i}\right)=1$; otherwise, $match_{t}\left(Train_{i},Test_{i}\right)=0$. The fitness of a chromosome represents the prediction accuracy obtained based on the corresponding feature selection. This value is continuously updated as the evolution progresses. Moreover, it is also used as an indicator to assess the quality of each chromosome. It provides a reference for subsequent genetic evolution. A better chromosome is more likely to be chosen for crossover.

**Step 4.** Apply genetic operators to derive new offspring

After a new generation is generated, the max fitness value searched for in the previous generation may be changed. As mentioned above, these genetic operators, including chromosome selection, crossover, and mutation, are intended to help generate new chromosomes. The selection operator determines whether a chromosome should be kept or eliminated depending on its fitness value. Chromosomes with a higher fitness value are more likely to survive. For crossover and mutation, the probabilities should be defined in advance.

**Step 5.** Repeat Step 3 and Step 4 until the stopping rule is met

Step 3 and Step 4 are iteratively executed until the stopping rule is satisfied. By the time that the expert system terminates the evolution based on the stopping rule, an optimal solution will be generated. This solution contains the finally selected features, which are most useful for the prediction of new cases and optimization of the weighting of features in the system.

**Step 6.** Evolution is completed

After genetic evolutions, the system outputs selected features. 

However, system configuration affects the solution performance of the GA framework and further reduce the robustness of the system.

To enhance the robustness of the proposed system, a GA and the Taguchi method are integrated into the expert system.

The Taguchi method [50] is utilized to optimize the system. It uses an orthogonal array and a signal-to-noise ratio (SN ratio) to help expert systems find an optimal system configuration. The advantage of using an orthogonal array is that it can significantly reduce the total number of runs of the experiment to slash the time cost, whereas the advantage of using an SN ratio is that the quality of the system can be measured. The Taguchi method designed for the system consists of three processes: firstly, set up the parameters of system; secondly, define the levels of each parameter; finally, generate the orthogonal array for the system. An example is given as follows. Assume that there are three parameters, and each parameter has three levels. For a full factorial experiment, the system needs to perform 27 experiments, which is quite time-consuming. Using the Taguchi method, this system generates an orthogonal array and needs to perform only nine sets (i.e., $L_{9}\left(3^{3}\right)$) of experiments to obtain a reliable solution. In this way, while the system execution time is being drastically reduced, the system quality can also be ensured.

After the configuration training is completed, the system will measure the mean-square error (*MSE*) of the expected results based on the data from each run. The *MSE* value has a smaller-the-better characteristic. It is expressed as follows:

(2) $$MSE=\frac{1}{n}\times \overset{n}{\underset{i=1}{\sum }}{\left(A_{i}-P_{i}\right)}^{2}\;$$

where n is the number of observations in the test data, $P_{i}$ is the predicted value for the $i^{th}$ observation, and $A_{i}$ is the actual value of the $i^{th}$ observation.

After measuring the *MSE* value for each run, the system will estimate the *SN* ratio for each configuration. The *SN* ratio has a larger-the-better characteristic. It is expressed as follows:

(3) $$SN=-10\times \log\left(\frac{1}{m}\overset{m}{\underset{j=1}{\sum }}\frac{1}{MSE_{j}{}^{2}}\right)\;$$

where m is the number of repetitions for each configuration, and $MSE_{j}$ denotes the result of the jth run.

Finally, the system will obtain the robust configuration $P_{q}$ $\left(q=1,2,\ldots ,Q\right)$ with the highest total *SN* ratio from all the runs. It can be expressed as follows:

(4) $$R\left(configuration\;P_{q}\right)=\mathrm{Max}\;SN_{P_{q_{k\left(k=1,2,\ldots ,K\right)}}}\;$$

where K is the number of levels for each parameter.

Details on data collection and performance of the system are provided in Section 3.

## 3. Results

### 3.1. Data Collection

Microsoft Excel 2016 (https://www.microsoft.com (accessed on 30 June 2022)) was installed as the runtime environment to implement the program. The proposed system was built using Evolver version 8.2 (https://www.palisade.com (accessed on 30 June 2022)) to process the genetic operations in the training. VBA (Visual Basic for Application) programming was also integrated to build the proposed expert system.

**Figure 4:**
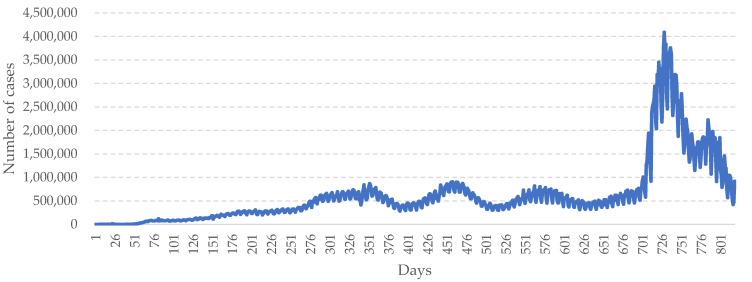
Daily new confirmed COVID-19 cases worldwide.

The data used in this study are real COVID-19 data reported from around the world. The data comprise the statistics of the pandemic provided on WHO’s official website (https://covid19.who.int (accessed on 30 June 2022). According to the statistics, the number of daily confirmed cases remains very high and COVID-19 is still severely spreading across the world (see Figure 4). A large number of infections have been caused since the outbreak of COVID-19. Daily infections of the virus peaked in the first quarter of 2022. Despite devotion to pandemic control, all governments around the world have been unable to resist the repeated growth of infections due to the virus’ continuous mutation and evolution. At present, hundreds of thousands of confirmed COVID-19 cases are being reported in many countries every day, indicating that the pandemic is still raging [3,4].

**Figure 5:**
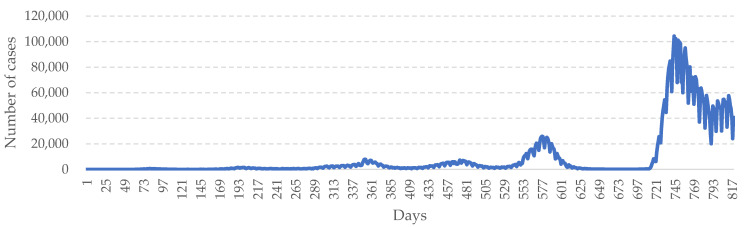
Daily new confirmed COVID-19 cases in Japan. (January 2020–April 2022).

Japan has been one of the hardest-hit areas for COVID-19 in Asia since 2020. More importantly, Japan decided to postpone the 2020 World Olympic Games in 2021. As shown in Figure 5, the number of confirmed cases in Japan peaked two times within one year. The case of Japan shows that it has experienced several ups and downs of the epidemic. In particular, under the invasion of the Omicron variant virus, Japan also reached the peak of the epidemic in March 2022. Thus, the spread of the epidemic in Japan has virtually become the focus of global attention. In this study, we chose Japan as the subject in the hope of performing an effective prediction of the epidemic trend in Japan. Real data from Japan were collected for data compilation and subsequent analysis. The data include daily statistics of confirmed COVID-19 cases in Japan from the outbreak of the pandemic.

**Figure 6:**
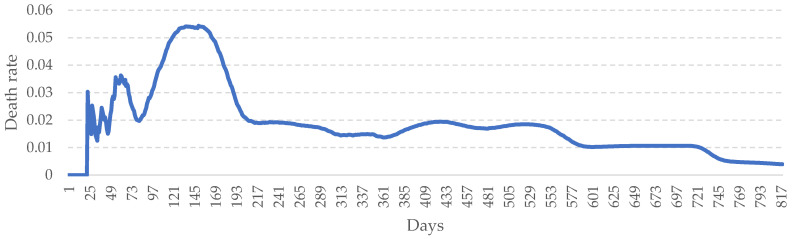
Daily death rate in Japan. (January 2020–April 2022).

This period spans from January 2020 to April 2022. In this period, the highest number of daily confirmed cases was 104,345, and the average number of daily confirmed cases was approximately 9080 with a standard deviation of 19,819. In addition, the death rate of COVID-19 in Japan was usually higher than 0.003. The highest number of daily death cases was 322, and the average number of daily death rate was approximately 0.018 with a standard deviation of 0.012 (see Figure 6). The historical peak of COVID-19 mortality in Japan occurred in the first year. Although the death rate has gradually decreased, the death toll remains high because the variant virus increases its susceptibility. It shows that reducing the mortality rate has become an important challenge for the government in Japan.

When a pandemic begins to spread in large numbers (such as COVID-19), governments are most concerned about the rapid rise in confirmed cases. In the post-epidemic era, governments would pay particular attention to issues relating to the reduction of the mortality rate, including the treatment of severe cases and how to provide effective treatments. Therefore, this study uses the death rate as an important evaluation index and classifies the epidemic situation into different alert states as a reference for healthcare policy makers.

The dataset comprises 819 days of COVID-19 data in Japan. We defined death rates for a daily number equivalent to or below 0.005 as low death rates (I), death rates between 0.005 and 0.01 as mid death rates (II), and death rates equivalent to or above 0.01 as high death rates (III). In the dataset, 83 cases were classified into the low death rate level (I), 34 cases into the mid death rate level (II), and 702 cases into the high death rate level (III). Death rate is defined as follows:

(5) $$\mathrm{Death}\;\mathrm{rate}=\frac{total\;deaths}{total\;confirmed\;cases}\;$$

In addition, this study uses demographic variables including population, population density, middle-aged population (middle age), population aged 65 or older (aged 65 older), population aged 70 or order (aged 70 older), cardiovascular disease death rate (CVD death rate), diabetes prevalence, hospital beds per thousand, and GDP per capital. The descriptive variables of COVID-19 collected in this study include new confirmed cases, hospital patients, ICU patients, people vaccinated, people fully vaccinated, and stringency index. These variables are used as the features of the COVID-19 dataset (as shown in Table 2). All the above variables are continuous variables.

### 3.2. Performance of the System

After data collection, we imported all the data into the system to train features through the GA framework. After training, the selected features could be used to predict the number of death cases over the next 120 days. The training dataset consists of data from 22 January 2020 to 31 December 2021. The validation dataset spans from 1 January 2022 to 21 April 2022.

To optimize training and system performance, using an appropriate system configuration is very important. Different configurations of parameters affect the performance of the system in finding a solution. Based on the Taguchi method [30], we adopted an orthogonal array and *SN* ratio to find the optimal set of parameters, including (1) $\mathrm{population}\times \mathrm{generation}\;\left(\mathrm{PS}\times \mathrm{GN}\right)$, (2) crossover rate (CR), and (3) mutation rate (MR).

As shown in Table 2, for each parameter, three different levels were adopted in the system. Considering the solution seeking time, the number of PS×GN was set as 20,000. Therefore, the system was designed to execute an $L_{9}\left(3^{3}\right)$ experiment, where 9 at the bottom indicates the number of experimental runs, 3 at the center stands for the number of levels, and 3 at the top represents the number of parameters.

The experimental runs were based on the three levels of each parameter. The *MSE* value in each configuration of parameter levels was measured three times (m=3) to obtain $MSE_{1}$, $MSE_{2}$, and $MSE_{3}$. The *MSE* function has a smaller-the-better characteristic.

After measuring *MSE*s, the system estimated the *SN* ratio for each configuration. Then, the system computed the results from all the runs of the experiment respectively.

Table 3 shows the *MSE*s and *SN* ratios from all of the runs of the experiment. As mentioned above, the *SN* ratio has a larger-the-better characteristic. The system summed the *SN* ratios from all of the runs of the experiment (as shown in Table 4) and selected the levels with the max sum. Consequently, the system obtained the configuration with the highest robustness of parameters as follows: PS = 400, GN = 50, CR = 0.75, and MR = 0.05. The average accuracy for the death rate level inference of the proposed system was 60%.

**Figure 7:**
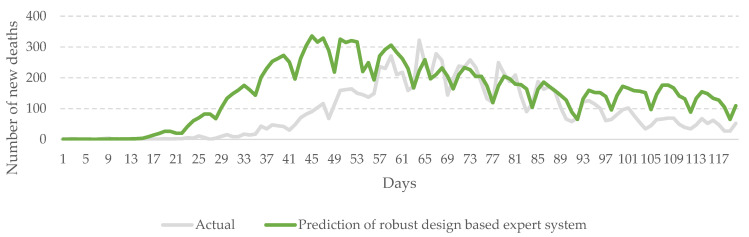
Prediction of robust design-based expert system. (January 2022–April 2022, in Japan).

After a robust configuration was obtained (including selected features), Japan’s epidemic data from 22 January 2020 to 31 December 2021 was imported into the system to predict the epidemic trend over the next 120 days. The results are shown in Table 4 and Figure 7. In addition, from the 15 features, 10 more representative ones were also selected, resulting in a selection rate of 67%.

It can be easily discovered from Table 5 that these features are mainly descriptive variables and demographic variables relating to COVID-19. As to the features associated with CVD death rate and diabetes prevalence, no significant difference was found between different epidemic levels. The gray line shows the actual data, while the green one represents the epidemic trend predicted by the proposed system (see Figure 7). The results indicate that the mean number of daily death cases of the proposed system was 151 persons with a standard deviation of 94 persons in this span of prediction. In the actual data, the mean number of daily death cases was 89 persons with a standard deviation of 84 persons. The time point of the increase in the death rate estimated by this system is slightly earlier than the actual data. Overall, it is quite close to the actual situation. A further comparison of the system’s prediction with the actual data was conducted.

It can be discovered that the prediction of the proposed system was close to the real situations. As shown in Figure 7, the system has captured the potential trend pattern of the outbreak. For example, there was an obvious increase in the number of death cases in Japan during the prediction period (i.e., 18 January 2022–22 February 2022). In the actual data, the number of daily death cases grew rapidly from 10 to 332 within four weeks. This abnormal surge was captured by the system effectively. In addition, the following trend in the epidemic situation was also predicted by the system. The results indicate that the performance of the proposed system is good.

**Figure 8:**
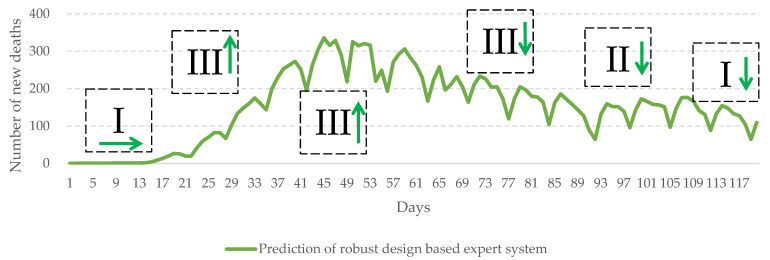
Monitoring levels for COVID-19 suggested by the system. (January 2022–April 2022, in Japan).

Furthermore, the robust design-based expert system can give a warning when the number of death cases is about to rise (as shown in Figure 8). For management authorities, this represents a practical and managerial implication concerning the formulation of measures.

For instance, when the epidemic is mild, the system suggests a monitoring level of I, meaning that healthcare authorities can maintain the current management policy. When the death rate grows higher than 0.01, the system will suggest a monitoring level of III. Healthcare authorities should: impose restrictions on the economic activity of certain industries and adopt crowd diversion measures for confirmed cases; require patients with mild illnesses to implement home quarantine, so as to reserve the medical capacity for patients in moderate or severe conditions; provide special care to patients in high-risk groups (including elderly and young children); and set up easily accessible PCR testing sites to instantly identify confirmed cases and provide medication. After the epidemic reaches its peak and begins to slow down through levels III and II with a decreasing death rate, the system suggests a monitoring level of I. Healthcare authorities are advised to progressively relax social management measures, lift the limitations on the economic activity of all industries, and also encourage people to conduct self-health management (as shown in Figure 8).

The monitoring levels suggested by the system based on dynamic changes in the pandemic cases can assist the authorities concerned to plan and deploy in advance. Therefore, the proposed system can be very helpful in practical applications where it is used as a policy support system.

To explore whether different compositions of the training dataset would affect the system’s predictive performance, we created three training datasets respectively consisting of 100%, 95%, and 90% of the training data. For each training dataset, the training was carried out five times, and the *MSE* was measured. Finally, analysis of variance (ANOVA) was applied to test if there was any significant difference in prediction accuracy between datasets.

Table 6 shows the ANOVA results, which suggest that under the significance level of 0.05, there was no significant difference in prediction accuracy between the training results with 5% and 10% reduced data.

## 4. Discussion

This study applied a robust design-based expert system with a feature selection learning mechanism to predict the possible trends of a pandemic disease. This system was designed to combine the strengths of the GA framework and the Taguchi method. In the case of Japan, the experimental results indicate that the proposed system can successfully capture the possible trends of COVID-19. The predicted results are close to the actual situations, meaning that the system’s capability is assured. With a unique design, the system can deliver good performance and stable quality. The prediction results of the system may be helpful for the government when estimating the death rate of the virus. Healthcare authorities can also employ the system to support formulation of epidemic management policies and allocation of medical resources.

Building an effective expert system is not easy [41,43]. This type of system usually requires repetitive tests, which are very time-consuming but do not guarantee the robustness of its results [44]. From the experimental results, we can find that the robust design has demonstrated its outstanding efficacy in improving the expert system. The orthogonal array design has significantly reduced the total number of runs of the experiment. The *SN* ratio can correctly reflect the differences between system configurations to further ensure the prediction quality of the system.

We also found that although the GA architecture is suitable for learning feature selection when performing an expert system experiment, the amount of the training dataset must be sufficient to obtain good results. In addition, due to the selection, crossover, and mutation mechanisms designed in the GA framework, the evolution of each feature in the chromosome can be evaluated accurately. This is key to the success of the feature selection expert system. Hence, the GA framework can achieve a good performance.

The proposed system can signal an alert when there is a rise in the level of death cases, which means that the number of severe cases has risen too. This alert has an important management implication for healthcare management authorities. If we can capture an imminent increase in the domestic death of the epidemic, the government can make necessary deployments in advance [7,8]. This way, the government’s use of medical resources can be more effective, and its responses to the pandemic can also be more timely. Thus, the proposed system is effective in supporting epidemic management.

**Policy implications**: In fact, the government’s healthcare resources are limited. If changes in the number of severe cases or death cases in the country cannot be correctly assessed, the government’s deployment of medical resources will be affected first. For example, when the epidemic is slowing down, health authorities can allow more patients with mild symptoms to stay in the hospital for treatment and observation and also delay their discharge time. Conversely, if the epidemic becomes more serious (i.e., the number of death cases grows rapidly), health authorities need to deploy ahead of time and change the management policies for mild and severe patients. For instance, when medical resources are insufficient, some countries may advise patients with a mild illness to stay at home to avoid medical collapse. Take Japan as an example. The repeated changes in the epidemic situation have put tremendous pressure on the government’s patient care policy. This highlights the importance of situational judgment. Therefore, the government’s primary task in COVID-19 management is to get hold of changes in the death rate in its territory and then make immediate adjustments of health care policies according to the epidemic trend. This kind of timely and situation-based response is conducive to the deployment of medical resources and patient management.

In the present, many countries have adopted policies that favor living with the virus. However, the spread and mutation of COVID-19 still pose a high threat. For example, despite the release of EUA-approved vaccines, many countries still experience large numbers of breakthrough infections after widespread vaccination. This phenomenon shows that epidemic control cannot rely solely on EUA-approved vaccines. All countries should be cautious in monitoring, isolation, and social management of confirmed cases. In the meantime, how to effectively get hold of the daily number of death cases becomes crucial and is an important task for governments when building an epidemic management information system. It determines whether governments can monitor the epidemic development in real time and adjust the degree of relaxation or contraction of medical resources at any time, so as to control the rate of virus transmission within the limits of national resources. Therefore, effective epidemic predictions with an appropriate support system can contribute greatly to epidemic control.

**Management implications**: In this study, the features considered in the prediction of COVID-19 epidemic trends consisted of descriptive variables and demographic variables. From the dataset of Japan, the average prediction accuracy was 60%. The system shows better accuracy given three levels of alert state were considered (e.g., there are three levels which means the probability is 33.33%). In addition, 10 representative features were also selected, resulting in a selection rate of 67% with a reduced rate of 33%. According to the experimental results, among the descriptive variables, new confirmed cases, ICU patients, and people vaccinated are important factors affecting the mortality of COVID-19 in Japan. In comparison, hospital patients and stringency index are not critical to the prediction result.

Among the demographic variables, population, population density, hospital beds per thousand, middle age, aged 70 older, and GDP per capita are all critical to the mortality of COVID-19 in Japan. However, CVD death rate, diabetes prevalence, and aged 65 older are not significantly related to mortality.

From the above findings, we can infer that, in addition to the descriptive variables that healthcare units are more concerned about (e.g., people vaccinated), the demographic structure of a country also significantly affects the pandemic mortality. This suggests that governments should pay attention to the correlation between the country’s demographic structure and the important descriptive variables of COVID-19, because the mortality of the pandemic may increase significantly with increases in certain descriptive variables such as new confirmed cases and ICU patients.

Therefore, the interactions between the abovementioned features and the differences in their importance should be a research topic worthy of further research.

This study was subject to two main limitations. First, the descriptive variables of COVID-19 are aggregated statistical data, and the database is not classified by virus variants determined through gene sequencing. Second, the subject of this study was Japan but the spread of the epidemic varies from country to country. With a good predictive performance, the proposed system can be applied to predict the spread of new COVID-19 variants and explore the differences in death rate between different variants. Moreover, the differences in importance between features is also an interesting research issue. The system architecture of this study can be modified for the research of the above issues in the future.

## 5. Conclusions

Expert systems are increasingly used in important applications. Healthcare management organizations across the world are seeking more accurate methods to predict the spread of epidemics and support policy adjustment. Considering the tremendous impacts that COVID-19 has brought to the world and the need for healthcare authorities to build a highly adaptive prediction system, this study proposes an enhanced artificial intelligence prediction technique called the robust design-based expert system. The GA framework and Taguchi method are integrated into this system to optimize the performance of the system. The epidemic data in Japan are employed to develop a prediction system for COVID-19. The prediction accuracy of proposed system was 60%. In addition, the feature selection rate of the system was 67% with a reduction of 33%. The experimental results indicate that the proposed system is effective in predicting the epidemic trend over the next four months (about 120 days). The proposed system can be utilized to support the prediction of epidemic trends as well as the deployment of resources. Future researchers can apply this system to analyze the epidemic trends in countries with more serious outbreaks and further explore the differences between countries and what can be improved with regard to the application of this expert system.

## Figures and Tables

**Table 1:** Comparison of previous research on feature selection.

Method	Issue	Year	Reference
GA-based algorithm	Large set feature extraction	1989	[35]
GA-based algorithm	Diagnostic classification	2008	[36]
Comparative domain corpora	Product improvement	2014	[37]
Ant-colony-based algorithm	Fault diagnosis	2015	[38]
GA-based algorithm	Breast cancer diagnosis	2016	[39]
GA-based algorithm	Breast cancer diagnosis	2017	[40]
GA-based algorithm	Heart disease diagnosis	2019	[41]
Machine learning	Hypertension Detection	2021	[42]
Machine learning	Comparison of different classifier ensemble methods	2021	[43]
Machine learning	Prediction of osteoporosis	2022	[45]

**Table 2:** Levels of key parameters for system.

Parameter	Level 1	Level 2	Level 3
PS×GN	200 × 100	400 × 50	100 × 200
CR	0.5	0.75	1.0
MR	0.05	0.075	0.1

**Table 3:** The $L_{9}(3^{3})$ orthogonal array for system.

Experiment	PS×GN	CR	MR	$MSE_{1}$	$MSE_{2}$	$MSE_{3}$	*SN*
1	1	1	1	0.0006010890	0.0006444215	0.0006394203	−64.0492353808
2	1	2	2	0.0004591344	0.0013799845	0.0004216040	−65.6009508154
3	1	3	3	0.0004056524	0.0004205735	0.0004056524	−67.7348319371
4	2	1	2	0.0004388614	0.0005938607	0.0006411313	−65.4242838171
5	2	2	3	0.0006394203	0.0006298676	0.0005938010	−64.1511242272
6	2	3	1	0.0011535812	0.0006116809	0.0004205265	−64.8091223273
7	3	1	3	0.0004344587	0.0006110492	0.0004362107	−66.4448913140
8	3	2	1	0.0005938607	0.0005989994	0.0004195332	−65.7611583377
9	3	3	2	0.0006485800	0.0004341845	0.0004500635	−66.2389340652

**Table 4:** Sum of *SN* ratios.

	PS×GN	CR	MR
Level 1	−197.3850181333	−195.9184105119	−194.6195160458 *
Level 2	−194.3845303716 *	−195.5132333803 *	−197.2641686977
Level 3	−198.4449837169	−198.7828883296	−198.3308474783

* Selected level with the max sum of *SN*.

**Table 5:** Selected and unselected informative features of COVID-19 in Japan.

Feature	Selection
*Descriptive variables of COVID-19*	
New confirmed cases	Selected
Hospital patients	Unselected
ICU patients	Selected
People vaccinated	Selected
People fully vaccinated	Selected
Stringency_index	Unselected
*Demographic variables*	
Population	Selected
Population density	Selected
Cardiovasc death rate	Unselected
Diabetes prevalence	Unselected
Hospital beds per thousand	Selected
median_age	Selected
Aged 65 older	Unselected
Aged 70 older	Selected
GDP per capital	Selected

**Table 6:** ANOVA result for training datasets.

	Sum of Squares	Degree of Freedom	Mean Sum of Square	*F*-Test	*p* Value
Between groups	1.1043 × 10^−5^	2	5.52149 × 10^−6^	2.776	0.102
Within groups	2.38722 × 10^−5^	12	1.98935 × 10^−6^		
Total	3.49152 × 10^−5^	14			

## Data Availability

The raw data are available from the corresponding author on reasonable request.
